# Physiological condition infers habitat choice in juvenile sockeye salmon

**DOI:** 10.1093/conphys/coae011

**Published:** 2024-04-01

**Authors:** Daniella C LoScerbo, Samantha M Wilson, Kendra A Robinson, Jonathan W Moore, David A Patterson

**Affiliations:** Department of Resource and Environmental Management, Simon Fraser University, Burnaby, BC V5A 1S6, Canada; Fisheries and Oceans Canada, Science Branch, Cooperative Resource Management Institute, School of Resource and Environmental Management, Simon Fraser University, Burnaby, BC V5A 1S6, Canada; Earth2Ocean Research Group, Simon Fraser University, Burnaby, BC V5A 1S6, Canada; Fisheries and Oceans Canada, Science Branch, Cooperative Resource Management Institute, School of Resource and Environmental Management, Simon Fraser University, Burnaby, BC V5A 1S6, Canada; Department of Resource and Environmental Management, Simon Fraser University, Burnaby, BC V5A 1S6, Canada; Earth2Ocean Research Group, Simon Fraser University, Burnaby, BC V5A 1S6, Canada; Fisheries and Oceans Canada, Science Branch, Cooperative Resource Management Institute, School of Resource and Environmental Management, Simon Fraser University, Burnaby, BC V5A 1S6, Canada

**Keywords:** Pacific salmon, Oncorhynchus, smolt, migration, physiology, energetics, salinity preference

## Abstract

The amount of time that juvenile salmon remain in an estuary varies among and within populations, with some individuals passing through their estuary in hours while others remain in the estuary for several months. Underlying differences in individual physiological condition, such as body size, stored energy and osmoregulatory function, could drive individual variation in the selection of estuary habitat. Here we investigated the role of variation in physiological condition on the selection of estuarine and ocean habitat by sockeye salmon (*Oncorhynchus nerka*) smolts intercepted at the initiation of their 650-km downstream migration from Chilko Lake, Fraser River, British Columbia (BC). Behavioural salinity preference experiments were conducted on unfed smolts held in fresh water at three time intervals during their downstream migration period, representing the stage of migration at lake-exit, and the expected timing for estuary-entry and ocean-entry (0, 1 and 3 weeks after lake-exit, respectively). In general, salinity preference behaviour varied across the three time periods consistent with expected transition from river to estuary to ocean. Further, individual physiological condition did influence habitat choice. Smolt condition factor (K) and energy density were positively correlated with salinity preference behaviour in the estuary and ocean outmigration stages, but not at lake-exit. Our results suggest that smolt physiological condition upon reaching the estuary could influence migratory behaviour and habitat selection. This provides evidence on the temporally dependent interplay of physiology, behaviour and migration in wild juvenile Pacific salmon, with juvenile rearing conditions influencing smolt energetic status, which in turn influences habitat choice during downstream migration. The implication for the conservation of migratory species is that the relative importance of stopover habitats may vary as a function of initial condition.

## Teaser

Salinity preference experiments were conducted on sockeye salmon smolts at lake-exit, estuary-entry and ocean-entry. Smolt condition factor and energy density were positively correlated with salinity preference in the estuary and ocean outmigration stages. Our results suggest that smolt physiological condition influences migratory behaviour and habitat selection.

## Introduction

Migratory periods are challenging phases in an organism's life cycle, often corresponding with periods of high mortality (Dingle & Drake, 2007). The mass, synchronous movement of individuals over long distances is also costly and requires substantial energy reserves to fuel relocation, often requiring the use of stopover habitats to refuel (Murray & Fuller, 2015). Changes in environmental conditions along migration routes may require individual physiological adaptations in thermal regulation or osmoregulation (Ramenofsky & Wingfield, 2007). Further, migrations can increase exposure to predation, which requires not only energy to fuel escape, but also behavioural avoidance of novel predators in changing environments (Sabal *et al*., 2021). Whether or not a migration will be successful depends on an individual’s ability to procure behavioural and physiological adaptations prior to and during migration.

Animal migrations are an integral component of ecosystem, economic and food stability, and yet are increasingly threatened by loss of habitat and connectivity (Wilcove & Wikelski, 2008; Hardesty-Moore *et al.*, 2018). Pacific salmon (*Oncorhynchus spp.*) provide a striking example of animal migration, colouring inland streams every year as spawners return to natal habitat to reproduce and filling coastal areas with silver smolts every spring as they transit from freshwater to ocean rearing areas. Climate change, fishing, habitat disturbance and landslides are identified as some of the top factors affecting Pacific salmon survival (Chalifour *et al.*, 2022; DFO, 2022; Reid *et al.*, 2022). Hence, effective conservation of such migratory species requires an understanding of the link between habitat and fish health at multiple life stages to accurately predict how changes to the landscape and aquatic environment will impact migratory salmon (Horodysky *et al.*, 2015; Hodgson *et al.*, 2019; Lennox *et al.*, 2019).

Estuaries form a critical ecotone between freshwater rearing habitats and the ocean growing ecosystem for anadromous salmon (Healey, 1982; Seitz *et al.*, 2020). As juvenile salmon leave their freshwater rearing habitat and swim towards the ocean, estuaries provide a range of salinities, resources and shelter to support their physiological transition to salt water (Iwata & Komatsu, 1984), growth (Moore *et al.*, 2016; Morrice *et al.*, 2020) and refuge from predation (Bevelhimer *et al.*, 2020; Seitz *et al.*, 2020) and parasites (Plantalech Manel-La *et al.*, 2009). Use of estuary habitat is associated with greater life history diversity (Reimers, 1971; Simenstad *et al.*, 1982; Jones *et al*., 2014), and the quality of estuarine habitat is correlated to returning adult survival in certain populations (Magnusson & Hilborn, 2003; Meador and MacLatchy, 2014). Despite the apparent benefits of residing in estuaries, the time period in which juvenile salmon remain in an estuary varies greatly among and within populations (Arbeider *et al.,* 2023), with some individuals passing through estuaries in a matter of days (Carr-Harris *et al.*, 2015; Moore *et al.*, 2016), while others remain in the estuary for several months (Levy & Northcote, 1982, Birtwell *et al.*, 1987, Beamish *et al.*, 2016). This variation in habitat use suggests that there may be underlying differences in individual physiology or behaviour that determine the importance of estuaries as stopover habitat for juvenile salmon.

Multiple aspects of physiological condition are associated with migration success in juvenile salmon and thus could influence use of estuaries. Smolt size and condition factor (K) may determine survival through the freshwater and early marine migration, as they are strong indicators of swim performance (Glova & McInerney, 1977; Taylor & McPhail, 1985a, 1985b) and contribute to migration speed at early ocean entry (Beacham *et al.,* 2014; Freshwater *et al.*, 2019). Juvenile salmon that are smaller and in poor condition are at greater risk of predation (Jeffries *et al.*, 2014; Osterback *et al.*, 2014; Tucker *et al.*, 2016). Underlying physiology, body size and differences in stored energy likely play an important role in survival through the migratory period (Wilson *et al.*, 2022). Downstream migration in smolts is active, and feeding opportunities are uncertain, thus there is speculation that smolts rely largely on stored energy from the rearing habitat to fuel migration to marine environments (Stefansson *et al.*, 2003; Quinn, 2005; Clark *et al.*, 2016). Stored energy is derived primarily from lipids, such as triglycerides, as well as proteins (Brett, 1995). The amount of stored energy at any given life stage determines the potential for growth and the likelihood of survival in fish (Larsen *et al.*, 2000; Post & Parkinson, 2001; Weber *et al.*, 2003) and can provide greater ecological and physiological relevance than traditional length or weight indices of fish condition (Trudel *et al.*, 2005; Minke-Martin *et al.*, 2018). In addition to size and stored energy, the ability of a smolt to maintain homeostasis as they enter the ocean depends on the production and activation of osmoregulating enzymes in the gills, most notable being Na^+^-K^+^ ATPase (NKA) (Evans *et al.*, 2005). Activity levels of NKA are predictors of downstream migratory behaviour of juvenile brown trout (*Salmo trutta*) (Elsner & Shrimpton, 2018, Houde *et al.* 2019, Nielsen *et al.*, 2004), and are associated with smolt survival during migration to the ocean in Atlantic salmon (*Salmo salar*) (Stich *et al.*, 2015). While previous work has focused on individual physiological constraints to migration patterns, the interplay of smolt size, energetic stores and osmoregulation on habitat selection during downstream migration remains a key knowledge gap.

**Figure 1:**
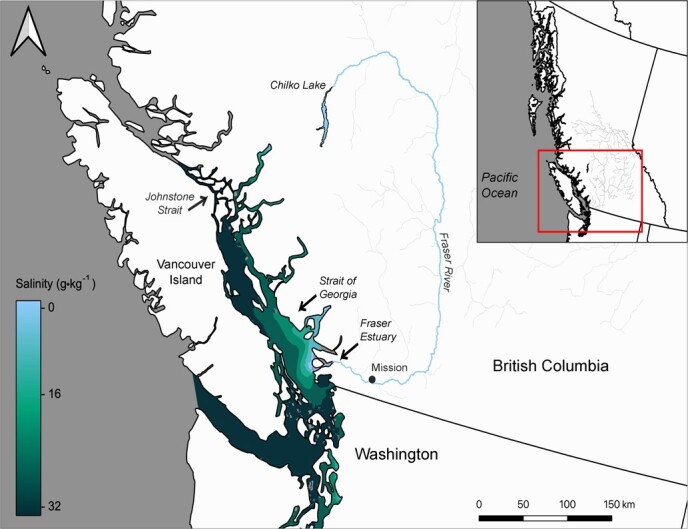
Map of Chilko sockeye salmon (*O. nerka*) migration route from Chilko Lake, down the Fraser River and entering the Strait of Georgia through the Fraser River Estuary.

Salinity preference is a commonly used metric to infer estuarine habitat choice for Pacific salmon smolts (Baggerman, 1960; Price & Schreck, 2003a; Stich *et al.*, 2016). Smolts swim through vertical and horizontal gradients of salinity when passing through estuaries and entering the ocean (Fig. 1) and salinity exposure can vary greatly even among a migrating cohort (Hedger *et al.*, 2008, Moser *et al.*, 1991; Plantalech Manel-La *et al.*, 2009). Experimental choice tests find that preference for saltwater increases throughout the outmigration period for juvenile salmon (Baggerman, 1960; McInerney, 1964). Previous work has linked fish condition to behaviour through salinity preference, where smolts that preferred full-strength seawater showed decreased indicators of stress (Price & Schreck, 2003b), disease (Price & Schreck, 2003a) and parasite infection (Webster *et al.*, 2007) when compared to those that preferred fresh water. The physiology underlying this behaviour has been attributed to seawater tolerance through hormonal control of gill NKA activity (Iwata & Komatsu, 1984; McCormick, 2013; Stich *et al.*, 2016), but no studies to date have investigated energetic predictors of salinity preference in conjunction with osmoregulation and fish size.

Here we examined potential linkages between juvenile salmon condition and salinity consistent with estuary and ocean habitat use. Specifically, we investigated the relationships between multiple metrics of Fraser River sockeye salmon smolt physiology and salinity preference. We performed salinity preference studies as an indication of habitat selection at three successive stages during the juvenile downstream migration period: (i) at the onset of downstream migration from the rearing lake, (ii) 6–10 days after outmigration from the rearing lake, when juveniles are expected to reach the Fraser River estuary (Clark *et al.*, 2016; Stevenson *et al.* 2019), and (iii) 21–24 days after outmigration, when juveniles are expected to have fully passed through the influence of the Fraser River estuary and reached the ocean in the Strait of Georgia and Johnstone Strait (Fig. 1; Beacham *et al.*, 2014; Clark *et al.*, 2016; Johnson *et al.*, 2019; Stevenson *et al.* 2019). We tested the hypothesis that salinity preference in migrating sockeye smolts is dependent on physiology and/or time since lake-exit. Previous work on sockeye salmon found that saltwater preference increases over migration time since lake-exit (Baggerman, 1960; McInerney, 1964). We predicted that saltwater preference would be lowest at lake-exit, intermediate at estuary-entry and highest at ocean-entry. Further, we predicted that saltwater preference within a migratory stage will be correlated to traits of increasing fish condition and saltwater tolerance: large size (length and weight), high body condition (K), high gill NKA activity and high densities of stored somatic energy (lipid and triglyceride percent). This work provides a mechanistic understanding between fish migratory behaviour and physiology, which links the condition of fish leaving the rearing environment to downstream effects on estuarine and coastal habitat use and population migration patterns.

## Materials and Methods

The focal population is sockeye salmon from Chilko Lake, located 650 river kilometres north of the Fraser River estuary, at an elevation of 1174 m, in the interior of British Columbia, Canada (Fig. 1). Outmigrating yearling sockeye salmon smolts were collected from Chilko Lake between 22:00 and 4:00 from 30 April to 1 May 2019, during the peak outmigration period (Macdonald *et al.*, 2020). Smolts were collected by dip net at a smolt fence and transferred to a riverside transport tank with continual flow-through of aerated river water. In total, 263 smolts were collected for salinity preference experiments. All fish collection, holding and experimentation protocols were reviewed and approved by the Animal Care Committee at Simon Fraser University (Protocol number 1238B-17).

Behavioural experiments were conducted on smolts at three time intervals during their downstream migration period, representing their lake-exit, estuary-entry and ocean-entry outmigration stages (0, 1 and 3 weeks after lake exit, respectively; see Clark *et al.*, 2016; Stevenson *et al.* 2019). Ocean entry here is defined as near–full-strength seawater that is beyond the freshwater influence of the Fraser River. To visualize the salinity gradient that migrating smolts are exposed to in Fig. 1, monthly average sea surface (0.5-m depth) salinity values were obtained from the SalishSeaCast Nucleus for European Modelling of the Ocean (NEMO) model (Soontiens *et al.*, 2016; Soontiens and Allen, 2017) for May 2019*.*

A random subset of smolts (*n* = 45) was selected to undergo lakeside salinity preference experiments to measure salinity preference, energetic reserves and physiology immediately following outmigration (hereafter, lake-exit). The remaining smolts (*n* = 218) were transported to the ALCAN research facility at Simon Fraser University, Burnaby, BC, and housed in freshwater and natural photoperiod for the duration of the experimental outmigration period (4 weeks following lake-exit, 2–29 May 2019). Smolts were randomly placed in one of four 80-gal holding tanks, each with a maximum density of ~15 g m^-3^. Water quality in holding tanks was maintained at 8.4°C (95% confidence interval [CI] [8.25–8.48]), 98.6% dissolved oxygen (95% CI [98.04–99.10]) and <0.25 ppm ammonia. Water temperatures were within the range of downstream migration conditions (Macdonald *et al.*, 2020) and were low enough to prevent desmoltification (<10°C, Stefansson *et al*., 1998).

To reflect observations of wild migrating smolts, as well as to maximize the contrast of energetic condition throughout migration, wild-caught smolts were not fed during the 4-week holding period in fresh water. Smolts were also above the critical energy threshold that limits swim performance (Hvas *et al.*, 2021; Wilson *et al.* 2021), suggesting the ability to actively search for different salinities was not impaired. The decision to not feed is supported by the observation that the majority of migrating sockeye smolts caught near the mouth of the Fraser River in Mission, BC, have empty stomachs (Patterson, unpublished data, personal communication), indicating that feeding is limited during the freshwater migration stage. A variable proportion (20–40% from 2004 to 2009, 29% in 2014) of juvenile sockeye collected in marine trawls in the Strait of Georgia have empty stomachs (Beamish *et al.*, 2012; Neville *et al.*, 2016) and show gene expression patterns that suggest recent fasting (Houde *et al.*, 2019).

### Experimental design

**Figure 2:**
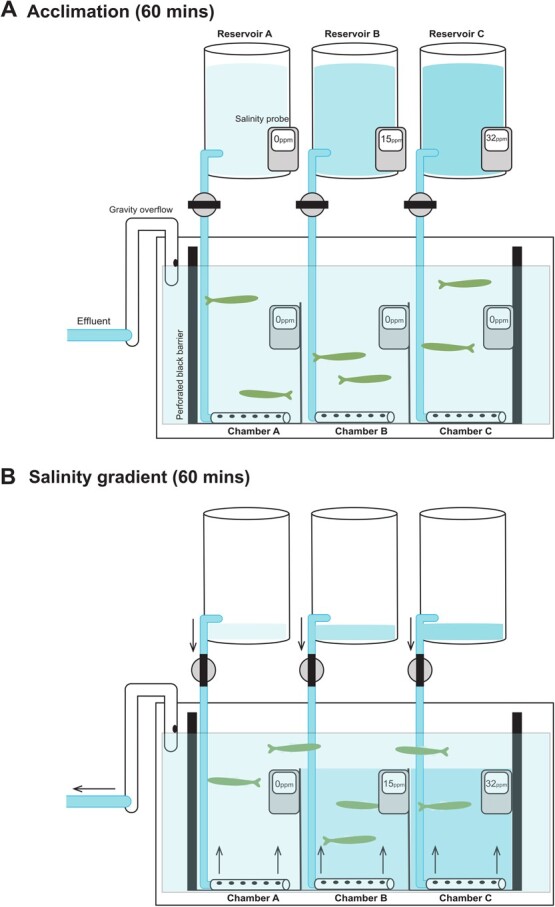
Experimental tank design to test for salinity preference of juvenile salmon. Smolts were transferred to aerated fresh water (A) for 1hour acclimation. A salinity gradient was then formed through bottom-fed fresh, brackish or salt water in respective chambers (B). Salinity preference as chamber occupancy was observed for 1 hour.

We performed salinity preference experiments to infer habitat choice among migrating smolts. The design of the experimental preference tank was modified from previous studies to test the preference for three salinities simultaneously within each migratory stage (Baggerman, 1960; McInerney, 1964; Price & Schreck, 2003b). Three 20 L glass aquaria were placed side by side within a 120 L aquarium (Fig. 2), dividing the larger aquarium into three chambers of equal size, while allowing for a 2inch gap above the dividing walls for fish to cross between chambers. The outside and bottom of the large aquarium was darkened with black plastic to minimize disturbance to fish during the experiment. The walls between chambers were left transparent to allow smolts to view conspecifics through the dividing walls. Smolts were tested in groups of six to allow for more natural schooling behavior of sockeye salmon along the horizontal plane (Katzman *et al.*, 2010, Tierney *et al.*, 2009; Webster & Dill, 2007), regardless of individual salinity choice. To distinguish individual smolts during the preference test, smolts were anesthetized and marked with coloured elastomer (Northwest Marine Technology ©). Tagging occurred the day before a trial to allow for a minimum of 18 hours for recovery. The tank was illuminated uniformly from above using an LED light and surrounded by shade cloth to prevent visual disturbance. A video camera (GoPro Hero 3 ©) was positioned facing the long side of the aquarium to record fish position within all three chambers throughout the experiment. In addition, an experimenter recorded fish position (chamber and depth) and behaviour every 10 minutes through viewing slots in the shade cloth.

To start the salinity preference trial, all three chambers were filled with fresh, aerated water (0.0–0.2 ppm, 8.0–9.7°C, 97.2–101.6% dissolved oxygen, *n*_trials_ = 42, Fig. 2A). Six smolts were randomly selected from holding tanks and transferred to the experimental aquarium, ensuring equally dispersed, randomized placement throughout the three chambers. The smolts were allowed to acclimate to the aquarium and explore the three chambers in fresh water for 1 hour. After the 1-hour acclimation period, a horizontal salinity gradient was imposed in the experimental tank by displacing the water of each chamber with either fresh (<2.0 ppt), brackish (14–16 ppt) or salt water (30–32 ppt) (Fig. 2B shows one of six possible orientations of the salinity gradient). The order of salinity of each chamber was randomized between each trial to control for any effect of chamber location or order on fish behaviour or movement. Each chamber was filled by separate water reservoirs that fed into the bottom of each chamber over approximately 5 minutes. Instant Ocean (Instant Ocean ©, Spectrum Brands, 3001 Commerce St., Blacksburg, VA 24060–6671) was added to filtered, aerated water to make brackish and salt water, and each were added to the respective reservoirs. The freshwater reservoir was filled with filtered, aerated water. The higher density salt and brackish water displaced the lower density fresh water upwards, forming a halocline; the upper freshwater layer served as a freshwater bridge in which the fish could move freely between chambers. Displaced fresh water was simultaneously drained from the aquarium using a gravity filtration system. Preliminary trials showed that the halocline was stable for over 2 hours. Salinity probes (Marine Salinity Waterproof Tester ©, HI98319, HANNA Instruments) were fixed within each chamber to monitor salinity and temperature throughout the duration of the experiments. Once the salinity gradient was imposed, fish position (chamber and depth) was recorded every 10 minutes for 1 hour. If a fish was in the freshwater layer above the halocline of a chamber, it was recorded as being in fresh water. In total, 44 experimental preference trials were undertaken. Eleven out of the 263 smolts tested were removed from behavioural analysis due to disturbances during experimental trials (e.g. technical failure of salinity gradient).

### Physiological sampling

Following preference trials, smolts were euthanized in tricaine methanesulfonate at a concentration of 400 mg L^−1^ (buffered with 800 mg L^−1^ NaHCO_3_) until fish operculum beating ceased and reflex response was absent (Neiffer & Stamper, 2009). Immediately after euthanization, gill tissue samples were clipped from the right gill arch and frozen on liquid nitrogen in SEI buffer (250 mM sucrose, 20 mM Na_2_EDTA, 50 mM Imidazole, pH 7.3) to preserve enzyme structure and function. Smolt carcasses were weighed and fork lengths measured before they were stored in whirlpacks and frozen on dry ice. Gill samples and carcasses were transported frozen on dry ice to the laboratory and stored at −80°C until analysis.

Gill NKA activity is a common metric to estimate saltwater preparedness in juvenile salmonids, where higher gill NKA activity indicates higher saltwater preparedness (McCormick, 2013). Gill NKA activity was measured following the enzymatic assay protocol from McCormick (1993; see Supplementary Material. Eight gill samples out of 263 produced high variation among replicates (Coefficient of variation [CV] > 20%) and were removed from analysis. To estimate the energetic status of individual smolts, lipids, water and ash were isolated and measured as proximate constituents following the Bligh and Dyer chloroform extraction method (Bligh & Dyer, 1959; Trudel *et al.*, 2005; Wilson *et al.*, 2021; see Supplementary Material). The energy density (ED) of each smolt was calculated from lipid (*f*) and protein (*p*) measurements using the following equation (Breck, 2008):

$$ ED=f{D}_f+p{D}_p $$

where *ED* is expressed in units kJ/g wet weight, *f* is the fraction of lipid measured per smolt (gg^-1^ wet weight), *D_f_* is the energy density of lipids reported for coho salmon (*Oncorhynchus kisutch*, 39.54 kJ g^-1^) (Brett & Groves, 1979; Crossin *et al.*, 2004; Higgs, 1979), *p* is the fraction of protein estimated per smolt (g g^-1^ wet weight) and *D_p_* is the energy density of protein reported for coho salmon (23.64 kJ g^-1^) (Crossin *et al.*, 2004; Higgs, 1979).

Triglycerides (TAG) are the major energy storage form of lipids in fish and have high ecological and physiological relevance as indicators of growth potential (Hakanson *et al.*, 1994; Lochmann *et al.*, 1995). A colorimetric method was used to determine percent TAG for each smolt (McGowan *et al.*, 1983; Weber *et al.*, 2003; see Supplementary Material). The percent ratio of TAG to lipid represents the density of TAG within lipid stores in individual fish. All replicate estimates of TAG density were within 20% CV. Measurements of TAG density >100% were removed from analysis (*n* = 2).

### Statistical analysis

To test for differences in length, mass and physiological condition between outmigration stages, group distributions were compared using a Type II Analysis of Variance (ANOVA) and Tukey *post hoc* test. All analyses were conducted using R version 4.0.2 and R Studio version 1.1.456 (R Core Team, 2018). Type II ANOVA and Tukey *post hoc* test were run using anova_test and tukey_hsd functions, respectively, in the rstatix package (version 0.6.0) (Kassambara, 2020b). Normality of variables was assessed visually using the ggqqplot function in the ggpubr package (Kassambara, 2020a), while heteroscedasticity of residuals was visually assessed using the plot function of the base R package (R Core Team, 2018).

Salinity preference was determined for 252 smolts. Salinity preference was calculated for individual smolts as the salinity of the chamber in which the smolt was observed >50% of the time after the salinity gradient was established (Larsson, 2005). If a smolt spent 50% of the experimental time in each of two chambers, salinity preference was determined as the salinity of the chamber occupied at the end of the trial. Fish that did not move between chambers throughout the acclimation and experimental stages were deemed non-exploratory and were not included in behavioural or physiological analyses. Non-exploratory smolts did not differ from exploratory smolts in physiological condition, nor did exclusion of non-exploratory smolts alter salinity preference results. A chi-squared test of independence was used to assess if there was an association between categorical variables of salinity preference and outmigration stage, using the function chisq.test in the R core package stats (Agresti, 2007; R Core Team, 2018).

The salinity preference experiment was designed to limit the choice of occupation to three chambers of increasing salinity, thus ordering the dependent variable as freshwater, brackish water or saltwater preference. Ordinal logistic regression assumes that the dependent variables are inherently ordered, the absence of multicollinearity between independent variables and proportional odds between each pair of outcome groups (Bilder & Loughin, 2014). Due to high collinearity among proximate constituents of somatic energy, energy density and condition variables, each variable was run as a separate predictor of salinity preference. The assumption of proportional log odds was tested for each model following Bilder and Loughin (2014) (Suppl. Table S1). The only exception was predicting salinity preference by NKA activity in the lake-exit group; only one smolt chose salt water, and that smolt displayed high NKA activity; when tested, this resulted in a predicted logit of negative infinity. Although this variable violates the assumption of proportional odds, it was included in the model for the lake-exit group as it reflects the true biological nature of the data, and transformations failed to be useful. When predicting salinity preference by NKA activity in the estuary and ocean outmigration groups, and across all three outmigration groups, the proportional odds assumption holds. The function polr in the R package MASS (Venables & Ripley, 2002; Ripley, 2020) was used to predict the ordinal outcome of salinity preference by measurements of smolt physiological condition for each outmigration stage separately. The assumption of normality and homoscedasticity of variables and residuals was verified via visual assessment for all models. All variables were standardized prior to model selection.

**Table 1:** Smolt physical and physiological condition throughout each outmigration stage of salinity preference experiments: lake-exit (1 day after lake-exit), estuary-entry (6–10 days following lake-exit), and ocean-entry (21–24 days following lake-exit).

Variable	Mean within outmigration stage (SD)			*n*	Intercept	*β*	*SE*	*P*
	Lake-exit	Estuary-entry	Ocean-entry					
Fork length (mm)	87.53 (5.46)	85.44 (6.32)	84.19 (4.93)	115	87.25	-0.149	0.059	**0.01**
Wet mass (g)	4.92 (0.90)	4.50 (0.96)	3.95 (0.81)	115	4.93	-0.045	0.009	**<0.001**
K	0.73 (0.05)	0.71 (0.06)	0.65 (0.04)	115	1.17	-0.004	0.001	**<0.001**
Water density, % (g g^-1^ wet weight)	79.42 (3.57)	80.63 (1.06)	81.46 (0.96)	108	79.63	+0.086	0.022	**<0.001**
Lipid density, % (g g-^1^ wet weight)	3.09 (1.03)	2.34 (0.82)	2.08 (0.48)	108	7.67	-0.040	0.009	**<0.001**
TAG density, % (g g^-1^ lipid)	32.93 (18.44)	31.61 (19.64)	15.60 (7.75)	107	36.39	-0.899	0.174	**<0.001**
Protein density, % (g g^-1^ wet weight)	17.38 (3.33)	16.92 (0.67)	16.35 (0.76)	108	17.36	-0.046	0.020	**0.02**
Energetic density (kJ g^-1^)	5.33 (0.92)	4.93 (0.36)	4.69 (0.28)	108	5.25	-0.026	0.006	**<0.001**
Gill NKA activity (μmol ADP mg protein^-1^ hr^-1^)	8.63 (3.33)	8.34 (2.56)	8.36 (3.16)	109	8.48	-0.005	0.032	0.9

The average value for each variable by outmigration stage is reported with the standard deviation in parenthesis. Results are from linear regressions of each condition variable by day since lake outmigration. The slope (*β*) indicates the estimated rate of change in condition per day, and the intercept indicates the model estimate for condition at lake outmigration. Models that were significant to *α* = 0.05 are shown in bold (*P*).

Smolt physical and physiological condition declined throughout the non-feeding holding period, as expected. Over the outmigration window, smolts declined in mass, K, whole-body percent lipid, whole-body percent protein, whole-body percent TAG and ED (Table 1). There was no change in gill NKA. To account for the decline in smolt condition (mass, K, lipid and protein stores) within the 4week holding time, residuals of the linear model of smolt condition by day of year were used as relative smolt condition to predict salinity preference across outmigration stages. The residuals of smolt condition by date thus account for daily changes in physiological and physical condition under the assumption that no feeding occurred during the downstream migration to the ocean. Smolts that were tested at lake-exit were on average longer (mean = 88 mm fork length, *SD* = 5 mm) than smolts tested at ocean-entry (mean = 84 mm, *SD* = 5 mm, Table 1). Although smolts were randomly sampled by netting from holding tanks, there was likely a handler-induced capture bias via the ease of catching larger fish versus smaller fish. To account for this size bias, fork length was included in all iterations of the full model. Models were selected by the second-order bias correction of Akaike’s Information Criterion (AIC_c_), recommended for comparison of models of sample sizes where *n* per *K* ≤ 40. AIC_c_ values were calculated with the function confset in the R package AICcmodavg (Burnham *et al.*, 2011; Mazerolle, 2020). The top model represents the most parsimonious model that minimizes information loss and overfitting.

## Results

Salinity preference behaviour varied over time (migration stage), consistent with the natural transition from river to estuary to ocean (Table 2). Salinity preference and outmigration stage were more strongly correlated than expected by chance (*χ^2^* = 17.52, *df* = 4, *P* < 0.01). At the onset of lake outmigration, the majority of smolts preferred fresh water (83%), and very few smolts selected brackish (13%) or salt water (4%). A shift in salinity preference occurred at the estuary-entry stage, where brackish water preference (37%) became equal to freshwater preference (37%), and saltwater preference increased to 26% of smolts. This general trend of preference held when tested at the ocean-entry stage, 2 weeks later, when 43% of smolts preferred fresh water, 31% preferred brackish, and 26% preferred salt water.

**Table 2:** The number of sockeye salmon smolts observed to prefer fresh water, brackish water or salt water at each stage of downstream migration: lake-exit (1 day after lake-exit), estuary-entry (6–10 days following lake-exit), and ocean-entry (21–24 days following lake-exit).

Outmigration stage	Number of fish by preferred salinity								
	Fresh water			Brackish			Salt water		
	**Obs.** (*SD*)	Exp.	Res.	**Obs.** (*SD*)	Exp.	Res.	**Obs.** (*SD*)	Exp.	Res.
Lake-exit	**25** (*0.05*)	15.39	2.45	**4** (*0.13*)	8.61	-1.57	**1** (*0.19*)	6.0	-2.04
Estuary-entry	**16** (*0.09*)	22.06	-1.29	**16** (*0.1*)	12.34	1.04	**11** (*0.08*)	8.6	0.82
Ocean-entry	**18** (*0.13*)	21.55	-0.76	**13** (*0.14*)	12.05	0.27	**11** (*0.10*)	8.4	0.90

*χ2 = 17.52, df = 4, P < 0.01*

The mean standard deviation of the proportion of time in preferred chamber is reported in brackets. The expected number of fish to reside in each chamber by chance (Exp.) was found by the product of the sum of fish in each salinity and the sum of fish in each outmigration stage, divided by total sample size of exploratory fish (*n* = 115). Residuals (Res.) are reported as the squared difference between the observed (Obs.) and expected (Exp.), divided by the expected number of fish to select either fresh water, brackish water or salt water in each migration stage using the Chi-squared test of independence.

Smolt physiology predicted salinity preference behaviour through the outmigration period from lake-exit to ocean-entry. All top models of salinity preference included physiological variables of osmoregulation and energy stores (Table 3). The top models of smolt behaviour across all stages of migration (lake-exit to ocean-entry) included gill NKA activity, TAG density and the migratory stage (lake-exit, estuary-entry or ocean-entry; Suppl. Table S2). The top model for smolts tested at estuary-entry included K, gill NKA activity and TAG density (Suppl. Table S3). At ocean-entry, the variables that contributed to the most parsimonious model were energy density and gill NKA activity (Suppl. Table S4). The only exception to this was at lake-exit, where the majority of smolts preferred fresh water regardless of physiology (Suppl. Table S5).

**Table 3:** Salinity preference model summaries for smolts tested within the estuary-entry, ocean-entry and all outmigration stages: lake-exit (1 day after lake-exit), estuary-entry (6–10 days following lake-exit) and ocean-entry (21–24 days following lake-exit).

Outmigration stage	Predictor	*β*	*SE*	*t* value	OR	2.5% CI	97.5% CI	*P*
Estuary-entry	K	22.90	8.47	2.71	2.96	1.43	9.29	**0.01**
	Gill NKA activity (μmol ADP mg protein^-1^ hr^-1^)	-0.00	0.14	-0.03	0.99	0.5	1.95	0.98
	TAG density, % (g g^-1^ lipid)	-0.04	0.02	-1.93	0.48	0.23	1.01	**0.05**
Ocean-entry	Energetic density (kJ g^-1^)	3.27	1.46	2.25	2.49	1.12	5.52	**0.02**
	Gill NKA activity (μmol ADP mg protein^-1^ hr^-1^)	0.20	0.11	1.82	1.86	0.95	3.61	0.07
All stages (lake-exit to ocean-entry)	Resid.(TAG density ~ DOY)	-0.01	0.01	-0.42	0.91	0.61	1.38	0.67
	Gill NKA activity (μmol ADP mg protein^-1^ hr^-1^)	0.14	0.07	1.92	1.51	0.99	2.29	**0.05**
	Fork length (mm)	-0.06	0.04	-1.56	0.71	0.46	1.09	0.12
	Estuary-entry	2.19	0.60	3.65	8.91	2.75	28.85	**<0.001**
	Ocean-entry	1.96	0.61	3.21	7.07	2.14	23.3	**<0.01**

In the lake-exit outmigration stage, the null model was selected as having the lowest AICc, and so it is omitted from this table. Models were selected by lowest AIC_c_. ORs and 2.5% and 97.5% CIs are shown for coefficients scaled by 1 *SD* increase/decrease of each explanatory variable. The *t* value shows the Wald statistic. Models that were significant to *α* = 0.05 are shown in bold (*P*).

Stage of outmigration had the strongest effect on smolt salinity preference, after accounting for variation in gill NKA activity and TAG density. Smolts tested during estuary-entry showed increased preference for brackish or salt water (*β* = 2.19, *SE* = 0.60, *P* < 0.001, Table 2), where the odds of choosing brackish or salt water increase by 8.9 times compared to those tested at lake-exit (95% CI [2.75–28.85]). Likewise, smolts tested during ocean-entry showed increased preference for brackish or saltwater (*β* = 1.96, *SE* = 0.61, *P* = 0.001), where the odds of choosing brackish or salt water increase by 7.1 times compared to those tested at lake-exit (95% CI [2.14–23.30]). Gill NKA activity was positively associated with increased salinity preference across the outmigration period but the effect was weak (*β* = 0.14, *SE* = 0.07, odds ratio [OR] = 1.51, 95% CI [0.90–2.29], *P* = 0.05). Accounting for TAG density increased model fit across outmigration stages, but TAG density alone was not a significant predictor of salinity preference.

**Figure 3:**
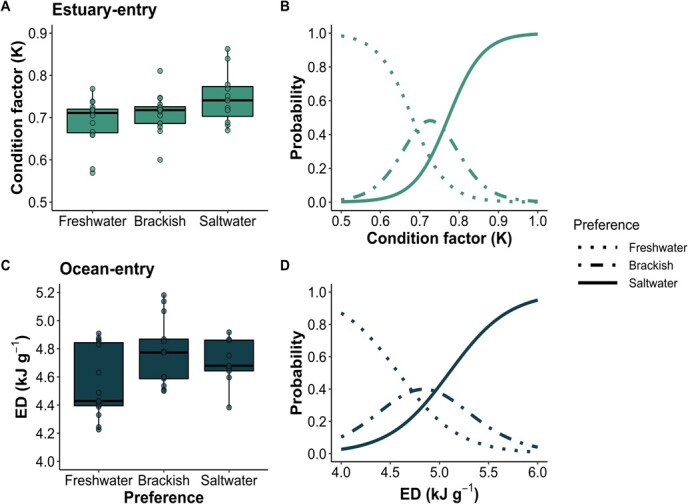
Distributions of fish condition variables that significantly contributed to top models predicting salinity preference at estuary entry (A) and ocean entry (C). Boxplots show the differences in K at estuary entry (A) and energy density at ocean entry (C) for fish that showed preference for fresh water, brackish water and salt water. Using top models, the probabilities of preference of fresh water (dotted line), brackish (dot and dash line) or salt water (solid line) were calculated over a range of values of smolt physiology at estuary entry and ocean entry. In the estuary model (B), probabilities were calculated for values of Fulton’s K while holding other variables constant at their respective mean values (M_NKA_ = 8.3 μmol ADP mg protein^-1^ hr^-1^, M_TAG_ = 31.6 g g^-1^ lipid). In the ocean model (D), probabilities were calculated for values of ED while holding gill NKA activity constant at the mean value (M_NKA_ = 8.4 μmol ADP mg protein^-1^ hr^-1^).

Condition factor and ED best explained smolt behaviour for estuary and ocean stages, respectively. In the estuary outmigration stage, higher values of K increased the likelihood of preference for brackish or salt water (*β* = 22.90, *SE* = 8.47, *P* = 0.01, Table 3), after accounting for gill NKA activity and TAG density. For every 1 *SD* increase in K (*SD* = 0.06), the odds of choosing brackish or salt water (≥15 ppm) increase by 2.96 times (OR = 2.96, 95% CI [1.43–9.29], Fig. 3). In the ocean outmigration stage, smolts with higher densities of somatic energy (ED) showed increased preference for brackish or salt water (*β* = 3.272, *SE* = 1.46, *P* = 0.02), after accounting for gill NKA activity. For every 1 *SD* increase in energy density (*SD* = 0.28 kJ g^-1^), the odds of choosing brackish or salt water (≥15 ppm) increase by 2.49 times (OR = 2.49, 95% CI [1.12–5.52], Fig. 3D).

## Discussion

Coupling behavioural experiments with physiological measurements, we found that behaviour is both dependent on the stage of the smolt outmigration (i.e. the duration of time since lake-exit), as well as the physiological condition of the migrating smolt. We show that the behavioural habitat preference of sockeye salmon smolts is dependent on physiological condition, providing a better understanding of how migrations of animals depend on both individual physiological and behavioural adaptations. Research on the salmon smolt migration has focused either on behaviour (Beamish & Neville, 2016; Clark *et al.*, 2016; Furey *et al.*, 2016) or physiology (Houde *et al.*, 2019; Stefansson *et al.*, 2012; Wilson *et al.*, 2021), but research that incorporates the interplay of the two has the opportunity to provide greater ecological relevance than either focus alone. Interestingly, the key physiological factors that best describe smolt behaviour, K and ED, change in importance throughout the smolt outmigration window. This dynamic connection between physiological condition and habitat choice during migration period could help explain annual or spatial variability in the importance of specific habitats, such as estuary use.

At different stages of the smolt outmigration, different physiological variables were more important for predicting salinity preference behaviour, K and ED at estuary-entry and ocean-entry, respectively**.** The trend in salinity preference observed suggests that smolts of lower physical (K) and energetic (ED) condition may choose to remain in the less saline waters of the estuary, rather than continue into full-strength seawater. Lower K was also found to be the strongest predictor of poor swim performance in migrating Chilko sockeye smolts (Wilson, *et al.* 2021). Studies that found a relationship between salmonid smolt size and estuarine residence (Levings *et al.*, 1986; Moore *et al.*, 2016; Chalifour *et al.*, 2020; Sawyer, *et al.* 2023), migration speed (Freshwater *et al.*, 2019) and coastal residency (Freshwater *et al.*, 2019) did not report K or energetics. We found that fork length and mass failed to predict salinity preference, suggesting K is a more integrative measure that can inherently encompass variation across a suite of important physiological variables, such as morphometrics, muscle mass and energy reserves, each of which contribute to survival and performance when passing through the complex environment of the estuary.

Our results also support the use of K as an accessible indicator of smolt migratory status. To measure K in the field, observers are only required to measure fork length and mass, and fish can be measured non-lethally with anaesthesia. By taking measurements of K for a sample of a migrating population of smolts, the proportion of smolts either passing through, residing in estuaries or continuing on to coastal waters can be predicted. As a link between growth before ocean entry and early marine survival has been seen in other salmonids (Bond, *et al.* 2008; Norrie *et al.* 2022), this can help inform survival estimates of key stocks for stock assessment. Predictions on the proportion of migrating smolts passing through or stopping over in estuaries can also assist in conservation efforts for informing the timing and degree of seasonally important habitat along salmon migration corridors.

The shift in importance from a morphology (K) to an energy (ED) metric of condition at ocean entry as a predictor of habitat preference is likely related to the diminishing energetic reserves at this late stage in outmigration. Migrating Atlantic smolts are shown to be ‘energy deficient’ from fresh water to early ocean entry (Stefansson *et al.,* 2003; Stefansson *et al.*, 2012), while coho smolts sampled at early ocean entry show gene expression indicative of fasting (Houde *et al.*, 2019), and Chinook (*Oncorhynchus tshawytscha*) smolts show evidence of lipid and TAG depletion in the ocean when compared to those in the estuary (MacFarlane & Norton, 2002). Without significant feeding during active downstream migration, smolts rely on the energy that was stored before leaving the rearing habitat. In this study, smolts tested at ocean-entry had an average energy density of 4.69 kJ g^-1^. This is close to the 3.47 kJ g^-1^ critical energy threshold for sustained swimming observed in Chilko sockeye smolts (Wilson *et al.*, 2021). Increased energy is also required for osmoregulation in marine environments and may explain the heightened importance of energy stores at this stage of migration. The metabolic rate of juvenile salmon is higher in saline water compared to fresh water (Maxime 2002, Morgan and Iwama, 1991, Wagner *et al.* 2006). At ocean entry, the amount of stored lipids is positively correlated to survival of juvenile Chinook (Burrows, 1969; Higgs *et al.*, 1992). At this late stage of the smolt migration, energetic reserves would be of heightened importance and may influence smolt behaviour to seek environmental conditions that impose the least energetic cost, such as seeking isotonically neutral (9–12 ppt; Brett, 1979) estuary waters with feeding opportunities.

Surprisingly, osmoregulation was not the strongest predictor of salinity preference in smolts. The stage of outmigration, K and energy stores (TAG or ED) were better at predicting behaviour than variation in gill NKA activity. Generally, we found that smolts with higher gill NKA activity were more likely to choose brackish or salt water, supporting observations in Atlantic smolts (Stich *et al.*, 2015, 2016). This trend was independent of other physiological parameters, such as size, physical condition and energy stores, and remained stable while in fresh water. The independence of size and osmoregulatory function is also noted in previous work on Chilko sockeye smolts (Bassett *et al.*, 2018) and Atlantic smolts (Whalen *et al.*, 1999). Work on coho salmon found that larger smolts develop higher gill NKA activities than smaller smolts, but this relationship diminishes throughout the outmigration window (Zaugg & McLain, 1972). While osmoregulatory function is an essential mechanism of migration in smolts, our findings suggest that salinity preference in habitat selection is more complex than simply determined by gill NKA activity.

The combined effect of physical and energetic condition with osmoregulation may have important implications to the use of downstream habitats that vary in salinity and foraging opportunity. Our results suggest that smolts of higher K with higher energetic stores and gill NKA activity will choose to enter the ocean habitat earlier than smolts of lower K, stored energy and NKA activity. This implies that smolt physiology should be assessed using multiple traits of condition specifically related to the adaptations required for the life stage in question. As seen in this study, one trait or condition metric does not encompass the full breadth of physiological status in smolts with respect to understanding critical movement behaviours such as salinity preference.

Migration phase had the strongest effect on the likelihood a smolt would prefer brackish or salt water, after accounting for smolt physiology. This trend of increasing salinity preference over time since lake exit is consistent with the course of outmigration expected in the wild, as exposure to salinity will increase from river to estuary to ocean, and provides support for the experimental approach taken. In this study, smolts were captured after lake-exit and held in a laboratory setting lacking variable environmental cues associated with outmigration behaviour such as changes in water temperature (Clark *et al.*, 2016, Sykes & Shrimpton, 2010; Zydlewski *et al.*, 2005), increasing photoperiod (Baggerman, 1960; McCormick *et al.*, 2002) and changing river flow rate (Katzman *et al.*, 2010; Sykes & Shrimpton, 2010). Despite the lack of environmental variability, we found that preference for brackish and salt water increased as expected at the timing of estuary-entry and was maintained through the period corresponding to ocean-entry. Changes in salinity preference of smolts throughout downstream migration are known to be conserved even in laboratory conditions (Baggerman, 1960; Otto & McInerney, 1970; Stich *et al.*, 2016). This temporal dependence of migration behaviour, observed without environmental cues, is mainly attributed to physiological changes associated with osmoregulation (Stich *et al.*, 2016; Sykes and Shrimpton, 2010; Zydlewski *et al.*, 2005). In support of this, we found that salinity preference behaviour was best explained when specific physiological variables were included with outmigration stage as predictors.

The estuary environment encompasses a suite of biophysical gradients that a migrating smolt experiences, such as food abundance, predation risk, temperature, depth, flow rate, turbidity and salinity (Plantalech Manel-La *et al.*, 2009). A salinity gradient is a simplified way to represent an estuary, and choice experiments are a simplified way to infer habitat choice. Future work should attempt combinations of multiple variables of the estuarine environment, such as turbidity, temperature and salinity preference. One of the larger assumptions of this study was that smolts do not feed during the downstream migration period, and although supported by some field observations of empty stomachs in migrating wild smolt (Beamish *et al.*, 2012; Neville *et al.*, 2016; Wilson *et al.*, 2021) and gene expression indicative of fasting at ocean entry (Houde *et al.*, 2019), the effects of starvation and migration stage in this experiment are inherently coupled. An effect of feeding on salinity preference behaviour is worth investigating further and could incorporate other behavioural metrics such as consumption rate in different salinities to show growth potential in regards to habitat choice within estuaries and coastal regions (see Webster & Dill, 2007). Comparing salinity preference changes with fish condition for salmon populations with longer estuary residence, such as ocean-type sockeye, Chinook and coho (Chalifour *et al.*, 2019, 2020), would show if our findings hold across life history types.

Our results provide novel evidence on the temporally dependent interaction between physiology, behaviour and migration in juvenile Pacific salmon. The large degree of intra- and inter-population variation in energetic condition at lake-exit in smolts (Wilson *et al.*, 2021, 2022) highlights the potential for carryover effects from parr to smolts that has implications for annual and population-level variability
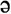
in estuary use (O’Connor *et al.*, 2014; Freshwater *et al.*, 2019; Hodgson *et al.*, 2020). Changes in rearing lake conditions, such as changes in primary productivity or fry densities, can change both the number and size distribution of sockeye smolts at lake-exit (Bradford *et al.*, 2000). As juvenile migratory behaviour is linked to physiological condition, and physiological condition is determined by growth conditions within the rearing habitat, estuaries may be of heightened importance for wild juvenile salmon in years of poor freshwater growth conditions. The importance of estuaries likely varies across years, populations and within a population cohort; therefore, the conservation of behavioural diversity in the use of estuaries can act as a buffer, enhancing the resilience of populations to environmental change (Flitcroft *et al.*, 2018). These findings support the growing body of evidence on the importance of conserving both rearing habitat for juvenile growth potential and estuarine habitat for smolt refugia before ocean-entry.

## Supplementary Material

Web_Material_coae011

## Data Availability

Physiological and behavioural data are available for download online at the DRYAD repository (https://doi.org/10.5061/dryad.dv41ns25p)
