# Supplementary material for: Physiological condition infers habitat choice in juvenile sockeye salmon
Source: Conserv Physiol. 2024 Apr 1;12(1):coae011. doi: 10.1093/conphys/coae011 (PMC10998697; doi:10.1093/conphys/coae011)
Supplement: Web_Material_coae011 [file web_material_coae011.pdf]

Supplementary Material

Physiological condition infers habitat choice in juvenile sockeye salmon

METHODS

Measures of Stored Somatic Energy

To estimate the energetic status of individual smolts, lipids, water and ash were isolated and measured as proximate constituents following the Bligh and Dyer chloroform extraction method (Bligh & Dyer, 1959). Sample percent lipid was calculated by taking the ratio of isolated lipid extracted from the homogenized tissue (g) to the weight of the sample used (g) and multiplying it by the ratio of the volume of chloroform + lipid used to dilute the homogenate (ml) to the volume of chloroform + lipid extracted to isolate the lipid (ml). Sample percent water was calculated by first taking the ratio of the dehydrated sample weight (g) to the homogenized sample weight (g) to get the percent moisture loss, and then taking the difference in percent moisture loss from 100. Sample percent carbon was calculated by the ratio of fully combusted ash sample weight (g) to the homogenized sample weight (g). Percent protein was estimated as the percent difference from the sum of percent lipid, water and carbon (Trudel et al., 2005). Energy data from four out of the 263 smolts analyzed were removed from further analysis due to high variance (CV>20%) among replicates for either lipid, carbon or water estimates. The energy density (ED) of each smolt was calculated from lipid (*f*) and protein (*p*) measurements using the following equation (Breck, 2008):

$$ED = fD_f + pD_p$$

where *ED* is expressed in units kJ/g wet weight, *f* is the fraction of lipid measured per smolt (g/g wet weight), D_f is the energy density of lipids reported for coho salmon (*O. kisutch*, 39.54 kJ g^{-1}) (Brett & Groves, 1979; Crossin et al., 2004; Higgs, 1979), *p* is the fraction of protein estimated per smolt (g g^{-1} wet weight), and D_p is the energy density of protein reported for coho salmon (23.64 kJ g^{-1}) (Crossin et al., 2004; Higgs, 1979).

Triglycerides (TAG) are the major energy storage form of lipids in fish and have high ecological and physiological relevance as indicators of growth potential (Hakanson et al., 1994; Lochmann et al., 1995). A colorimetric method was used to determine percent triglycerides for each smolt (McGowan et al., 1983; Weber et al., 2003). A sample of lipid extract was taken from the chloroform-lipid extraction, the chloroform was evaporated from the sample, and then a colorimetric assay was used to determine the concentration of TAG in each sample. The percent ratio of TAG to lipid was determined by multiplying TAG concentration (g mL^{-1}) of the sample by the total volume of chloroform used for lipid extraction and dividing by the mass of lipid extracted from the sample. The percent ratio of TAG to lipid represents the density of TAG within lipid stores in individual fish. Absorbance was read at 540 nm in a FLUOStar Omega © optic fluorescence spectrometer. The percent ratio of TAG to lipid represents the density of TAG within lipid stores in individual fish. All replicate estimates of TAG density were within 20% CV. Measurements of TAG density above 100% were removed from analysis (n = 2).

Osmoregulatory Preparedness: Gill NKA Activity

Gill NKA activity is a common metric to estimate saltwater preparedness in juvenile salmonids, where higher gill NKA activity indicates higher saltwater preparedness (Bassett et al., 2018;

Elsner & Shrimpton, 2018; Stich et al., 2016). Gill NKA activity was measured following the enzymatic assay protocol from McCormick (1993). A FLUOStar Omega © optic fluorescence spectrometer was used to measure the rate of oxidation of NADH to NAD+, a proxy for ATP hydrolysis. Reagent quality was checked prior to each assay by running a standard curve of ADP consumption in the absence of NKA, in which the acceptable range was -0.17 to -0.2 mOD $nmol^{-1}$. Each gill sample was measured in triplicate and was coupled with a sample that was inhibited by 0.5 mM ouabain. Assay mixtures of homogenized gill tissue samples and reagents were measured for change in absorbance at 340 nm and 25˚C. The linear slope of ATP hydrolysis was calculated over 10 minutes for each sample in Omega BMG Labtech Software © 5.10 R2. The slope of ATP hydrolysis was then corrected by dividing by the measured ADP extinction coefficient standard curve ($\bar{x}$ = 20.754 mOD $nmol^{-1}$ NADH, SE = 0.140, n = 3). The enzyme activity of each well was then scaled by the corresponding concentration of protein from each tissue sample using the BCA Protein Assay Kit © (Product No. 23225 from Pierce P.O. Box 117, Rockford, Illinois 61105). Protein assays were measured in triplicate, with bovine serum albumin as the standard, read at 25˚C and 550 nm absorbance. The final metric of NKA activity was determined as the difference between uninhibited and ouabain-inhibited rates of ATP hydrolysis in units of micromoles ADP per milligrams of protein per hour. Eight gill samples out of 263 produced high variation among replicates (CV>20%) and were removed from analysis.

TABLES

Suppl. Table 1. Proportional log odds test for explanatory variables of ordinal logistic regression models following Bilder & Loughin (2014).

Outmigration stage	Explanatory variables	Res. Diff.	*df*	*p*-value
Lake-exit	FL	3.237500089	1	0.07197
	K	0.175744686	1	0.675056
	lipid	0.010841176	1	0.917073
	protein	0.187446291	1	0.665051
	ED	0.166765946	1	0.683002
	TAG	0.144204366	1	0.704137
Estuary-entry	FL	0.179573096	1	0.67174
	K	0.298451226	1	0.584855
	lipid	1.433589057	1	0.23118
	protein	0.005569505	1	0.94051
	ED	1.328275539	1	0.249112
	TAG	1.416196057	1	0.234031
	NKA	1.490824557	1	0.222089
Ocean-entry	FL	1.165391115	1	0.28035
	K	0.101414945	1	0.750138
	lipid	1.114342244	1	0.29114
	protein	0.185719012	1	0.666504
	ED	1.553210987	1	0.212662
	TAG	0.00793452	1	0.929022
	NKA	1.011233094	1	0.314608

Suppl. Table 2. Model selection for predicting smolt salinity preference by physical and physiological condition across all outmigration stages (lake-exit, estuary-entry, and ocean-entry). Models were selected by the second-order bias correction of Akaike's Information Criterion (AICc). Residuals of Fulton's condition factor (resid. K), triglyceride density (resid. TAG), lipid density (resid. lipid), protein density (resid. protein), and energetic density (resid. ED) were used as explanatory variables to account for daily decline during the 4 week non-feeding holding period. Fork length was included in all iterations of the full model to account for an observed size-bias.

Candidate Model	AICc	ΔAICc	Model Likeli.	AICc Wt	Least Likeli.	Cum. Wt
resid. TAG + NKA + outmig. stage + FL	196.83	0	1	0.58	-91.23	0.58
resid. lipid + NKA + outmig. stage + FL	199.03	2.2	0.33	0.19	-92.33	0.77
resid. ED + NKA + outmig. stage + FL	199.85	3.02	0.22	0.13	-92.74	0.9
resid. protein + NKA + outmig. stage + FL	200.39	3.56	0.17	0.1	-93.01	1
NKA + outmig. stage + FL	210.03	13.2	0	0	-98.88	1
resid. TAG + outmig. stage + FL	210.75	13.92	0	0	-99.24	1
resid. lipid + outmig. stage + FL	213.88	17.05	0	0	-100.81	1
resid. ED + outmig. stage + FL	214.01	17.18	0	0	-100.88	1
resid. protein + outmig. stage + FL	214.48	17.65	0	0	-101.11	1
resid. K + outmig. stage + FL	220.53	23.7	0	0	-104.14	1
NKA + FL	222.06	25.23	0	0	-106.97	1
outmig. stage	224.31	27.48	0	0	-108.1	1
FL + outmig. stage	224.44	27.61	0	0	-107.13	1
resid. TAG + FL	225.13	28.3	0	0	-108.5	1
resid. mass + outmig. stage	226.07	29.23	0	0	-107.94	1
resid. protein + FL	228.58	31.75	0	0	-110.23	1
resid. ED + FL	228.6	31.77	0	0	-110.24	1
resid. lipid + FL	228.63	31.8	0	0	-110.25	1
resid. mass + FL	230.08	33.25	0	0	-110.98	1
resid. K + FL	233.92	37.09	0	0	-112.9	1
FL	237.25	40.42	0	0	-115.59	1
1	239.22	42.39	0	0	-117.59	1

Suppl. Table 3. Model selection for predicting smolt salinity preference by physical and physiological condition in the estuary-entry outmigration stage. Models were selected by the second-order bias correction of Akaike's Information Criterion (AICc).

Candidate Model	AICc	∆AICc	Model Likeli.	AICc Wt	Least Likeli.	Cum. Wt
K + NKA + TAG	81.18	0	1	0.73	-35.31	0.73
K + TAG	84.48	3.31	0.19	0.14	-38.06	0.87
K + NKA + protein	88.61	7.44	0.02	0.02	-39.04	0.89
K + NKA + ED	88.88	7.71	0.02	0.02	-39.18	0.9
K + NKA + lipid	89.07	7.89	0.02	0.01	-39.27	0.91
TAG + NKA	89.1	7.92	0.02	0.01	-40.37	0.93
FL + NKA + TAG	90.19	9.01	0.01	0.01	-39.82	0.94
K + ED	90.4	9.23	0.01	0.01	-41.03	0.94
TAG	90.43	9.25	0.01	0.01	-42.11	0.95
K + lipid	90.46	9.29	0.01	0.01	-41.06	0.96
K + protein	90.53	9.35	0.01	0.01	-41.09	0.96
K + NKA	90.65	9.48	0.01	0.01	-41.16	0.97
mass + NKA + TAG	91.26	10.08	0.01	0	-40.35	0.98
FL + TAG	91.71	10.53	0.01	0	-41.67	0.98
K	92.39	11.22	0	0	-43.1	0.98
mass + TAG	92.53	11.35	0	0	-42.09	0.98
ED + NKA	93.82	12.64	0	0	-42.73	0.99
lipid + NKA	93.82	12.64	0	0	-42.74	0.99
protein + NKA	93.82	12.65	0	0	-42.74	0.99
FL + NKA + protein	94.41	13.23	0	0	-41.94	0.99
FL + NKA + lipid	94.45	13.28	0	0	-41.96	0.99
FL + NKA + ED	94.46	13.28	0	0	-41.96	0.99
protein	94.52	13.34	0	0	-44.16	0.99
ED	94.69	13.51	0	0	-44.24	0.99
lipid	94.69	13.52	0	0	-44.25	0.99
FL + protein	95.32	14.14	0	0	-43.49	0.99
FL + NKA	95.59	14.41	0	0	-43.63	1
FL + lipid	95.63	14.46	0	0	-43.65	1
FL + ED	95.69	14.52	0	0	-43.68	1
mass + NKA + protein	95.7	14.53	0	0	-42.59	1
mass + NKA + ED	95.72	14.54	0	0	-42.6	1
mass + NKA + lipid	95.73	14.55	0	0	-42.6	1
mass + protein	96.38	15.2	0	0	-44.02	1
mass + lipid	96.65	15.47	0	0	-44.15	1
FL	96.66	15.48	0	0	-45.23	1
mass + ED	96.66	15.48	0	0	-44.16	1
NKA	96.66	15.49	0	0	-45.23	1
1	97.36	16.18	0	0	-46.63	1
mass + NKA	97.71	16.53	0	0	-44.69	1
mass	98.47	17.29	0	0	-46.14	1

Suppl. Table 4. Model selection for predicting smolt salinity preference by physical and physiological condition in the ocean-entry outmigration stage. Models were selected by the second-order bias correction of Akaike's Information Criterion (AICc).

Candidate Model	AICc	ΔAICc	Model Likeli.	AICc Wt	Least Likeli.	Cum. Wt
ED + NKA	72.42	0	1	0.23	-32.01	0.23
K + NKA + ED	72.85	0.42	0.81	0.18	-31.11	0.41
mass + NKA + ED	73.15	0.73	0.69	0.16	-31.26	0.57
FL + NKA + ED	73.51	1.08	0.58	0.13	-31.44	0.7
lipid + NKA	74.86	2.44	0.3	0.07	-33.22	0.77
protein + NKA	75.87	3.44	0.18	0.04	-33.73	0.81
mass + NKA + lipid	76.02	3.6	0.17	0.04	-32.7	0.84
FL + NKA + lipid	76.07	3.65	0.16	0.04	-32.72	0.88
K + NKA + lipid	76.26	3.84	0.15	0.03	-32.82	0.91
FL + NKA + protein	77.08	4.66	0.1	0.02	-33.23	0.94
mass + NKA + protein	77.46	5.04	0.08	0.02	-33.42	0.96
K + NKA + protein	78.09	5.67	0.06	0.01	-33.73	0.97
TAG + NKA	78.44	6.01	0.05	0.01	-35.01	0.98
FL + NKA + TAG	79.67	7.24	0.03	0.01	-34.52	0.99
mass + NKA + TAG	80.21	7.79	0.02	0	-34.79	0.99
K + NKA + TAG	80.54	8.12	0.02	0	-34.96	0.99
ED	83.04	10.62	0	0	-38.41	1
NKA	83.58	11.16	0	0	-38.68	1
K + ED	84.29	11.86	0	0	-37.96	1
FL + NKA	85.05	12.63	0	0	-38.34	1
mass + ED	85.11	12.69	0	0	-38.37	1
FL + ED	85.18	12.76	0	0	-38.41	1
protein	85.37	12.95	0	0	-39.58	1
mass + NKA	85.48	13.06	0	0	-38.56	1
K + NKA	85.58	13.16	0	0	-38.61	1
lipid	86.08	13.65	0	0	-39.93	1
FL + protein	87.43	15.01	0	0	-39.53	1
mass + protein	87.47	15.05	0	0	-39.55	1
K + protein	87.52	15.1	0	0	-39.58	1
K + lipid	88.01	15.59	0	0	-39.82	1
FL + lipid	88.22	15.8	0	0	-39.93	1
mass + lipid	88.22	15.8	0	0	-39.93	1
TAG	88.69	16.27	0	0	-41.24	1
K + TAG	90.69	18.27	0	0	-41.16	1
FL + TAG	90.83	18.4	0	0	-41.23	1
mass + TAG	90.83	18.41	0	0	-41.23	1
1	94.57	22.14	0	0	-45.23	1
K	96.53	24.11	0	0	-45.17	1
FL	96.65	24.23	0	0	-45.23	1
mass	96.66	24.24	0	0	-45.23	1

Suppl. Table 5. Model selection for predicting smolt salinity preference by physical and physiological condition in the lake-exit outmigration stage. Models were selected by the second-order bias correction of Akaike's Information Criterion (AICc).

Candidate Model	AICc	∆AICc	Model Likeli.	AICc Wt	Least Likeli.	Cum. Wt
1	36.18	0	1	0.11	-16.02	0.11
K	36.53	0.36	0.84	0.09	-15.13	0.2
TAG	37.77	1.59	0.45	0.05	-15.74	0.25
lipid	37.95	1.77	0.41	0.05	-15.84	0.3
protein	38.08	1.91	0.39	0.04	-15.9	0.34
NKA	38.1	1.92	0.38	0.04	-15.91	0.39
K + TAG	38.14	1.97	0.37	0.04	-14.84	0.43
FL	38.23	2.05	0.36	0.04	-15.97	0.47
mass	38.26	2.09	0.35	0.04	-15.99	0.51
ED	38.31	2.13	0.34	0.04	-16.02	0.55
K + lipid	38.34	2.16	0.34	0.04	-14.93	0.58
K + protein	38.69	2.51	0.28	0.03	-15.11	0.61
K + ED	38.7	2.52	0.28	0.03	-15.11	0.65
K + NKA	38.72	2.55	0.28	0.03	-15.13	0.68
mass + TAG	39.47	3.29	0.19	0.02	-15.5	0.7
TAG + NKA	39.55	3.38	0.18	0.02	-15.54	0.72
lipid + NKA	39.62	3.45	0.18	0.02	-15.58	0.74
FL + TAG	39.95	3.77	0.15	0.02	-15.74	0.76
mass + lipid	40	3.83	0.15	0.02	-15.77	0.77
protein + NKA	40.07	3.89	0.14	0.02	-15.8	0.79
FL + lipid	40.1	3.92	0.14	0.02	-15.81	0.8
FL + protein	40.17	3.99	0.14	0.02	-15.85	0.82
FL + NKA	40.18	4	0.14	0.02	-15.85	0.83
mass + protein	40.23	4.05	0.13	0.01	-15.88	0.85
mass + NKA	40.27	4.1	0.13	0.01	-15.9	0.86
ED + NKA	40.29	4.11	0.13	0.01	-15.91	0.88
K + NKA + TAG	40.36	4.18	0.12	0.01	-14.82	0.89
FL + ED	40.4	4.22	0.12	0.01	-15.96	0.91
mass + ED	40.45	4.28	0.12	0.01	-15.99	0.92
K + NKA + lipid	40.52	4.35	0.11	0.01	-14.9	0.93
K + NKA + protein	40.93	4.75	0.09	0.01	-15.11	0.94
K + NKA + ED	40.94	4.76	0.09	0.01	-15.11	0.95
mass + NKA + TAG	41.41	5.23	0.07	0.01	-15.35	0.96
FL + NKA + TAG	41.77	5.6	0.06	0.01	-15.53	0.97
mass + NKA + lipid	41.81	5.63	0.06	0.01	-15.55	0.97
FL + NKA + lipid	41.82	5.65	0.06	0.01	-15.55	0.98
FL + NKA + protein	42.19	6.01	0.05	0.01	-15.74	0.99
mass + NKA + protein	42.3	6.12	0.05	0.01	-15.79	0.99
FL + NKA + ED	42.42	6.24	0.04	0	-15.85	1
mass + NKA + ED	42.52	6.34	0.04	0	-15.9	1

FIGURES

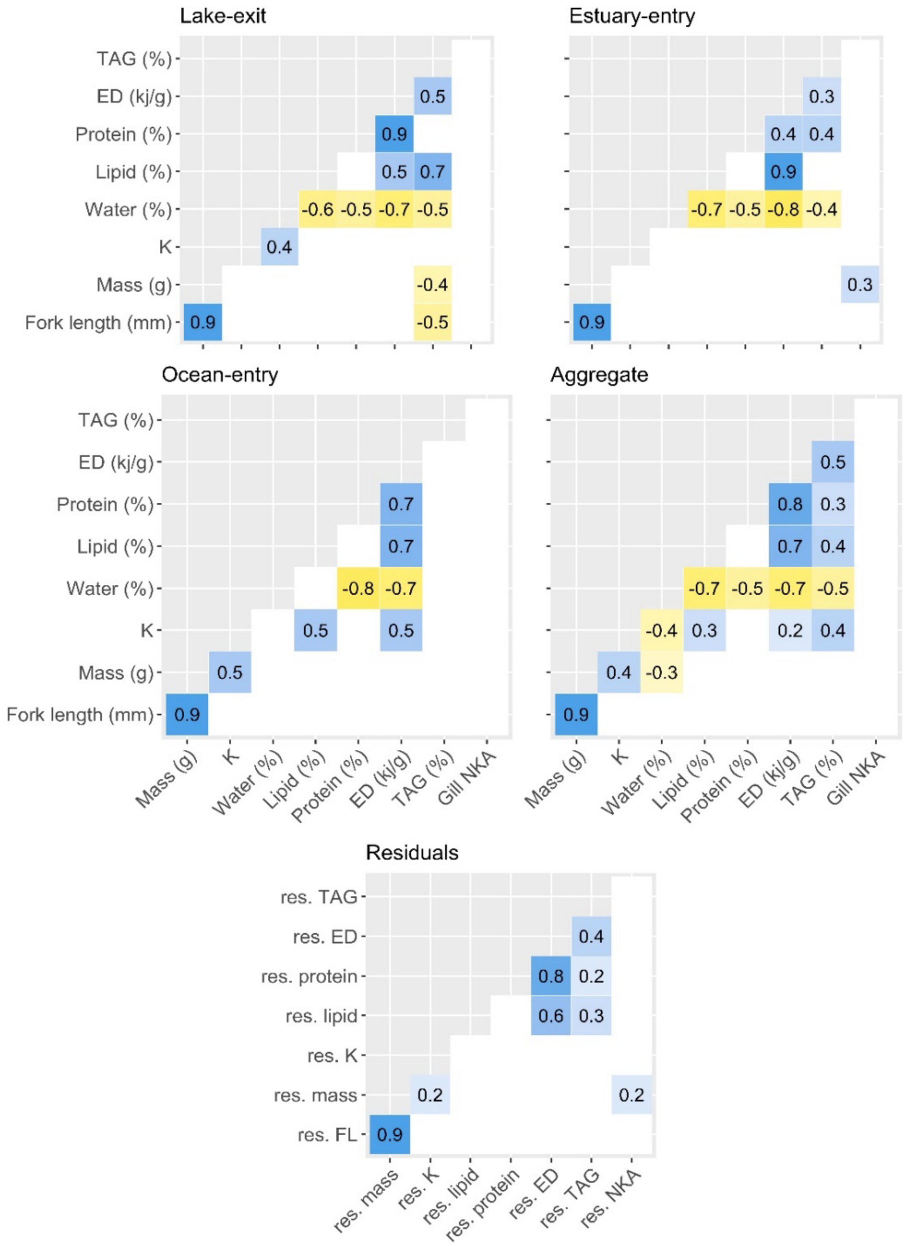

Suppl. Figure 1. Correlation matrix between physiological variables of Chilko sockeye smolts sampled throughout outmigration window: at lake-exit, estuary-entry, and ocean-entry. Variables from smolts sampled across all outmigration stages were pooled for aggregate correlations, and residuals of mass and energetic variables were corrected for linear temporal decline. Energetic density is denoted as ED, Fulton's condition factor as K, and gill NKA activity as NKA. Positive correlations are shown in blue and negative correlations in yellow. Color intensity is proportional to the correlation coefficients.

REFERENCES

Bassett, M. C., Patterson, D. A., & Shrimpton, J. M. (2018). Temporal and spatial differences in smolting among Oncorhynchus nerka populations throughout fresh and seawater migration. *Journal of Fish Biology*, *93*(3), 510–518. https://doi.org/10.1111/jfb.13678

Bligh, E.G. and Dyer, W. J. (1959). A rapid method of total lipid extraction and purification. *Canadian Journal of Biochemistry and Physiology*, *37*(8), 911–917.

Breck, J. E. (2008). Enhancing bioenergetics models to account for dynamic changes in fish body composition and energy density. *Transactions of the American Fisheries Society*, *137*, 340–356. https://doi.org/10.1577/t05-240.1

Brett, J. R., & Groves, T. D. D. (1979). Physiological energetics. *Fish Physiology*, *8*(6), 280–352.

Crossin, G. T., Hinch, S. G., Farrell, A. P., Higgs, D. A., Lotto, A. G., Oakes, J. D., & Healey, M. C. (2004). Energetics and morphology of sockeye salmon: Effects of upriver migratory distance and elevation. *Journal of Fish Biology*, *65*(3), 788–810. https://doi.org/10.1111/j.0022-1112.2004.00486.x

Elsner, R. A., & Shrimpton, J. M. (2018). Is the duration of the smolt window related to migration distance in coho salmon Oncorhynchus kisutch? *Journal of Fish Biology*, *93*(3), 501–509. https://doi.org/10.1111/jfb.13679

Hakanson, J. L., Coombs, S. H., & Ré, P. (1994). Lipid and elemental composition of sprat (Sprattus sprattus) larvae at mixed and stratified sites in the German Bight of the North-Sea. *Journal of Marine Science*, *51*, 147–154.

Higgs, D. A. (1979). Development of practical diets for coho salmon (Oncorhynchus kisutch), using poultry byproduct meal, feather meal, soybean meal and rapeseed meal as major protein sources. *Proceedings of the World Symposium on Finfish Nutrition and Fishfeed Technology*, *2*, 191–218.

Lochmann, S. Y. E., Gary, L., Taggart, G. T., & Maillet, K. T. F. (1995). Lipid class composition as a measure of nutritional condition in individual larval Atlantic cod (Gadus morhua). *Canadian Journal of Fisheries and Aquatic Sciences*, *52*, 1294–1306.

McCormick, S. D. (1993). Methods for nonlethal gill biopsy and measurement of Na+, K+-ATPase Activity. In *Canadian Journal of Fisheries and Aquatic Sciences* (Vol. 50, pp. 656–658). https://doi.org/10.1139/f93-075

McGowan, M. W., Artiss, J. D., Strandbergh, D. R., & Zak, B. (1983). A peroxidase-coupled method for the colorimetric determination of serum triglycerides. *Clinical Chemistry*, *29*(3), 538–542. https://doi.org/10.1093/clinchem/29.3.538

Stich, D. S., Zydlewski, G. B., & Zydlewski, J. D. (2016). Physiological preparedness and performance of Atlantic salmon Salmo salar smolts in relation to behavioural salinity preferences and thresholds. *Journal of Fish Biology*, *88*(2), 595–617. https://doi.org/10.1111/jfb.12853

Trudel, M., Tucker, S.Morris, J. F. T., Higgs, D. A., & Welch, W. W. (2005). Indicators of

energetic status in juvenile coho salmon and chinook salmon. *North American Journal of Fisheries Management*, *25*, 374–390. https://doi.org/10.1577/M04-018.1

Weber, L. P., Higgins, P. S., Carlson, R. I., & Janz, D. M. (2003). Development and validation of methods for measuring multiple biochemical indices of condition in juvenile fishes. *Journal of Fish Biology*, *63*, 637–658. https://doi.org/10.1046/j.1095-8649.2003.00178.x
